# Pain, Emotions, Interoception, and Bodily Sensations in Patients With Endometriosis Bodily Sensations in Patients With Endometriosis

**DOI:** 10.1002/ejp.70144

**Published:** 2025-10-06

**Authors:** Saara Pasternack, Juulia Suvilehto, Päivi Härkki, Oskari Heikinheimo, Reetta Sipilä, Eija Kalso

**Affiliations:** ^1^ Department of Anaesthesiology, Intensive Care and Pain Medicine Helsinki University and Helsinki University Hospital Helsinki Finland; ^2^ Pain Clinic, Neurocentre Helsinki University and Helsinki University Hospital Helsinki Finland; ^3^ Division of Cell and Neurobiology Linköping University Linköping Sweden; ^4^ AI Competence Centre Sahlgrenska University Hospital Sahlgrenska Sweden; ^5^ Department of Obstetrics and Gynaecology University of Helsinki and Helsinki University Hospital Helsinki Finland; ^6^ Current employer: National Centre for Pain Management and Research for Children and Adolescents, New Children's Hospital Helsinki University and Helsinki University Hospital Helsinki Finland; ^7^ SleepWell Research Programme, Faculty of Medicine University of Helsinki Helsinki Finland

## Abstract

**Background:**

Psychosocial aspects underlie and maintain persistent pain. Emotions have emerged as a target for psychological interventions in pain management. Our aim was to better understand the relationship between emotions and bodily sensations, including pain sensitivity, using two new approaches.

**Methods:**

110 patients with confirmed endometriosis and 110 age‐ and gender‐matched pain‐free controls completed computer‐based Bodily Sensation Maps for six basic emotions and a neutral emotional state, tactile, nociceptive, and hedonic sensitivities, as well as current and persistent pain. All participants also evaluated their current emotional experience of six basic emotions, depression, and anxiety, and answered the Brief Pain Inventory questionnaire; 102 endometriosis patients also answered the Multidimensional Assessment of Interoceptive Awareness questionnaire.

**Results:**

Endometriosis patients coloured in significantly larger painful areas on body maps and greater sensitivities to both nociceptive and hedonic sensations than did the pain‐free controls. The endometriosis patients reported more current fear than controls but did not differ from controls in the colouring in of basic emotions on the body maps. Emotional awareness was associated with higher pain intensity, and with more colouring for persistent pain. More trusting was associated with less affective interference and with less colouring for current pain. Less worrying and more trusting were associated with more colouring for hedonic sensitivity.

**Conclusions:**

Bodily sensation maps and multidimensional assessment of interoceptive awareness provide important information about the interface of emotions and pain. Our results suggest that a less worrying and a more trusting nature have a protective role in pain interference.

**Significance:**

Bodily emotions and interoceptive awareness associate with sensitivity to pain and should be addressed when targeting emotions in pain management.

## 1 Introduction

Endometriosis is an oestrogen‐dependent disease, where endometrial‐like tissue is located outside the endometrial cavity (Howard 2009). Endometriosis affects 10% of women of reproductive age (Zondervan et al. 2020). Characteristic symptoms include chronic pelvic pain, painful periods and intercourse, painful urination and bowel movements (Bulun et al. 2019). The multifactorial mechanisms of endometriosis‐associated pain include inflammatory and neurogenic factors, and peripheral and central sensitisation (Maddern et al. 2020; Zondervan et al. 2020).

Pain significantly affects the quality of life of women with endometriosis (Facchin et al. 2015; Jones et al. 2001), who often report lack of control of the disease and negative impact on self‐image (Jones et al. 2001). Endometriosis often disrupts the social, academic, and economic lives of young women (Bulun et al. 2019) and is linked to decreased emotional well‐being (Jones et al. 2001; Vitale et al. 2017). Psychosocial aspects underlie and maintain prolonged pain via pre‐existing vulnerability factors (Edwards et al. 2016). Protective factors have received less attention. The treatment of endometriosis has been mainly pharmacological and surgical, but the need for multidisciplinary treatments has been acknowledged (Ball and Khan 2020).

Interoceptive awareness is perceiving and processing information from inside the body, including physical sensations from internal organ functions (Mehling et al. 2009). The perception of heartbeat, satiety, respiration, or internal sensations related to emotions are examples of interoception. Changes in bodily states associated with external or internal stimuli are sensed via the interoceptive system and precede the conscious experience (Damasio and Carvalho 2013). Interoceptive awareness can be either adaptive or maladaptive (Hanley et al. 2017; Mehling et al. 2009). Interoceptive skills relating to the tendency “not to worry about unpleasant bodily sensations” predict lower pain intensity (Colgan et al. 2022) whereas increased worrying has been associated with increased pain perception (Ciaramella et al. 2023). Thus, the interoceptive system seems central in pain perception and hence as a target for pain management.

Bodily Sensation Maps (BSMs) give a visual form for the interoceptive information regarding pain, emotions, and other physical sensations (Nummenmaa et al. 2014). BSMs are constructed with the emBODY‐tool that has been studied with diverse cultures, ages, and conditions (Hietanen et al. 2016; Lloyd et al. 2021; Nummenmaa et al. 2014). A recent study found higher sensitivity for pain and tactile stimuli in relevant pain areas and reduced embodied experience of emotion in patients with persistent pain, compared with pain‐free controls (Ojala et al. 2023).

Our main aim was to study the connections between pain, interoception, and processing of bodily information, including emotions, in endometriosis patients. Secondly, we were interested in the differences between endometriosis patients and healthy participants in their ways of processing bodily information as measured with the emBODY‐tool. Our hypotheses are that endometriosis patients show reduced sensitivity to emotions and heightened sensitivity to nociceptive sensations compared with healthy controls, that interoceptive features related to orienting towards bodily sensations without anxiety or worry are associated with greater colouring in for emotions in the BSMs and less pain in the Brief Pain Inventory (BPI). This information could provide guidance for a more targeted multidisciplinary treatment of endometriosis‐associated pain.

## 2 Methods

This study is part of the subproject PROMPT (WP2) of IMI‐PainCare (https://www.imi‐paincare.eu/PROJECT/PROMPT/), an EU‐supported prospective multi‐centre observational study aiming to identify patient‐related factors associated with postoperative pain and recovery from surgery (clinicaltrials.gov, ID: NCT03834922). The current study is an add‐on sub‐study conducted at Helsinki University Hospital (HUH), focusing on the impact of emotions and interoception on pain in women with endometriosis. The study was approved by the Ethics Committee of Helsinki University Hospital (HUS/200/2020). All patients gave written informed consent.

### 2.1 Participants

#### 2.1.1 Patients

In total, 148 consecutive patients with pelvic or abdominal pain referred for surgery for confirmed or suspected endometriosis were recruited for the IMI‐PainCare‐PROMPT study at the Women's Clinic of HUH during November 2019—April 2021. Inclusion criteria were aged 18–80 years, planned surgical treatment, and informed consent to participate in the study. Exclusion criteria were inability to provide informed consent, cognitive decline, inadequate command of the Finnish language, secondary surgery due to complications or endometriosis surgery due to infertility.

#### 2.1.2 Healthy Pain‐Free Controls

For the analysis of BSMs, we selected age‐ and gender‐matched pain‐free controls (*n* = 110) from a larger population sample (Ojala et al. 2023), adjusted for the patient cohort of the current study. The female controls were algorithmically selected so that the age differential between the participant and control was the smallest possible while limiting to controls with low current pain. Only controls who answered “no” to the question of whether they had a persistent pain condition, reported acute pain intensity < 3 on a numerical pain scale (NRS 0–10) and < 3 on the Brief Pain Inventory (BPI) now and 24 h' mean, and had no history of significant menstrual or abdominal pain, were included.

### 2.2 Procedure

Approximately 1 week before surgery, patients completed a form with background information (gender, age, weight, height, educational level). They were asked to report the frequency of use of over‐the‐counter and prescribed analgesics, in addition to other medications affecting the central nervous system. Patients were asked about their history of repeated and/or very painful migraine, headache, back/shoulder pain, or joint/limb pain by choosing one of three options (“no”, “yes”, “yes but not during the last 6 months”). The patients were also asked about their history of abdominal or menstrual pain.

Following background information, the patients were asked to complete BPI and the BSMs, using the emBODY‐tool. When colouring in the BSMs, the patients were also asked to evaluate their current emotional state by rating how much they felt anger, fear, disgust, happiness, sadness, surprise, as well as anxiety and depression, on a scale from 0 (none) to 10 (the maximum possible). The patients also completed the Multidimensional Assessment of Interoceptive Awareness (MAIA) questionnaire.

For the age‐matched, healthy controls, only the answers to the emBODY‐tool along with background information, BPI, and evaluation of their current emotional states were available.

#### 2.2.1 Measures

The **Brief Pain Inventory (BPI)** (Cleeland and Ryan 1994) is used to assess the severity and interference of pain symptoms during the last 24 h. It comprises four questions about pain intensity (worst, least, average, now) and seven about pain interference on daily life (general activity, walking, work, mood, enjoyment of life, relations with others, sleep) on a 0–10 scale (for pain intensity: 0 = no pain and 10 = pain as bad as you can imagine; for pain interference 0 = no interference and 10 = interferes completely). Means for both subscales are calculated. Finally, the responder is asked about the degree of pain relief by analgesics. In this study, we used only sum scores for *Pain intensity* and *Pain interference* and calculated the sum scores for the interference subscales *Activity interference* (4 items: general activity, walking, work, sleep) and *Affective interference* (3 items: mood, enjoyment of life, relations with others). Cronbach's α (reliability) for BPI intensity was 0.85 and for BPI interference 0.93. Cronbach's α for BPI activity interference was 0.89 and for BPI affective interference 0.88.

The **Multidimensional Assessment of Interoceptive Awareness** (**MAIA**) (Mehling et al. 2012) is a self‐report measure to assess an individual's ability to detect and interpret internal bodily signals. Specifically, MAIA measures interoceptive sensibility, which is a subjective assessment of one's interoceptive awareness. MAIA has been developed to differentiate between anxious‐driven and mindful attention styles for interoceptive signals, an aspect that has been shown to be important in clinical settings. For a more comprehensive description, see Mehling (2016) & Mehling et al. (2012).

MAIA is a 32‐item self‐report measure, comprising eight separate dimensions of interoceptive awareness, as follows: *Noticing* (awareness of uncomfortable, comfortable, and neutral bodily sensations); *Not‐distracting* (tendency not to ignore or distract oneself from painful or uncomfortable sensations); *Not‐worrying* (tendency not to worry or experience emotional distress with sensations of pain or discomfort); *Attention regulation* (ability to sustain and control attention to body sensations); *Emotional awareness* (awareness of the connection between body sensations and emotional states); *Self‐regulation* (ability to regulate distress by attention to body sensations); *Body listening* (active listening to the body for insight); *Trusting* (experience of one's body as safe and trustworthy). Each dimension is measured on a 6‐point Likert scale, 0 indicating “never” and 5 “always”. Respondents are instructed to choose the number in each statement that would best describe their situation. Cronbach's α ranged from 0.70 ‐ 0.89 in the subscales, except for *Not‐distracting*, where a Cronbach's α of 0.32 was judged unacceptable. This subscale was therefore removed from the main analyses.

The **emBODY‐tool** (Nummenmaa et al. 2014) is a computer‐based, topographical method for collecting self‐reported embodied experiences of emotions and sensations. For a thorough description of the method, see Nummenmaa et al. (2014).

In the task, participants were shown a pair of two‐dimensional silhouettes of bodies and asked to colour the bodily regions where they would feel increased (hot colour) or decreased (cool colour) activity during a certain imagined stimulus. The data gathered with the emBODY‐tool was then combined into Bodily Sensation Maps (BSMs), with deactivations marked as −1, activations as 1, and uncoloured pixels as 0. Here, participants had three different categories of colouring task (emotions, pain, and sensory sensitivities) with multiple imagined stimuli in each category. The stimuli were shown one at a time, with instructions for the task category shown before the first task of each category. The categories were shown in a fixed order but the stimuli within each category were randomised for each participant. Patients completed the colouring tasks with iPad Pro tablets (Apple Inc) while the controls used their own devices.

First, participants were shown a front outline of a human body and asked to colour those areas where they experienced increasing or decreasing activation when experiencing a specific emotion. Only the name of emotion was shown for each task, with no further instruction to induce the emotion, or for imagining a situation where that emotion would be experienced. The six basic emotions specified were sadness, happiness, anger, surprise, fear, and disgust, as well as a neutral emotional state. In the other two categories of task, participants were shown the front and back outlines of a human body and first asked to colour in the areas for their current and persistent pain. After this, they were asked to colour in where they could easily feel even a light touch (*tactile sensitivity*), areas that they typically experienced as particularly sensitive to pain (*nociceptive sensitivity*), and areas whose touching they found pleasant (*hedonic sensitivity*).

### 2.3 Statistical Analyses

The data analysis was conducted with R 4.2.1. We analysed the data with non‐parametric tests, as most variables were not normally distributed. Group differences were explored with Mann–Whitney U tests and ꭓ^2^ tests for continuous and categorical variables, respectively; Spearman's ρ was used for correlation of continuous variables; Cronbach's α was used for assessing the internal consistency of the questionnaires.

Statistical comparisons for BSMs were made using mass univariate two‐sample *t*‐tests (for emotion tasks) or two‐proportion z‐tests (for pain and sensitivity tasks). The resulting comparison maps were corrected for false discovery rates (FDRs), adjusted for the number of pixels within the silhouette outline. Only significantly different FDR‐corrected pixels are shown as different between the two groups. FDR correction was chosen for the comparison of BSMs because it is less stringent than the alternatives for large numbers of pixels. A more conservative multiple comparison correction (Holm‐Bonferroni) was used for other statistical comparisons.

When comparing the groups for the self‐evaluated emotions, depression and anxiety, as well as the number of coloured pixels across emotions and sensitivities in the body maps, we used two‐way within‐between‐subject robust ANOVA on trimmed means, using the bwtrim function (as implemented in the {WRS2} package v[1.1–6]) suitable for non‐normally distributed data. The means were trimmed 20% from both ends (cutting 20% from the upper and 20% from the lower ends of the distribution) to account for non‐normal distribution and outliers (Mair and Wilcox 2020). For each ANOVA, there were two levels of group (patient or control) and a variable number of levels of the task. We constructed the model with interactions each time. For complementary analysis, Mann–Whitney U test was used with Holm‐Bonferroni multiple comparison correction.

Correlation matrices between pain, self‐reported emotional experience, and bodily sensitivities in endometriosis patients and pain‐free controls were created with the rcorr function (as implemented in the {Hmisc} package v[5.2–3]). For the correlation matrix, Spearman's ρ was calculated for each pair of variables and *p*‐values were corrected for multiple comparisons using Holm‐Bonferroni method.

Finally, when comparing the MAIA scores between endometriosis patients and healthy participants (Mehling et al. 2012), as well as low back pain patients (Mehling et al. 2013), we used two‐sample *t*‐tests, since only the means of MAIA subscale scores were available for healthy participants and low back pain patients.

## 3 Results

**FIGURE 1:**
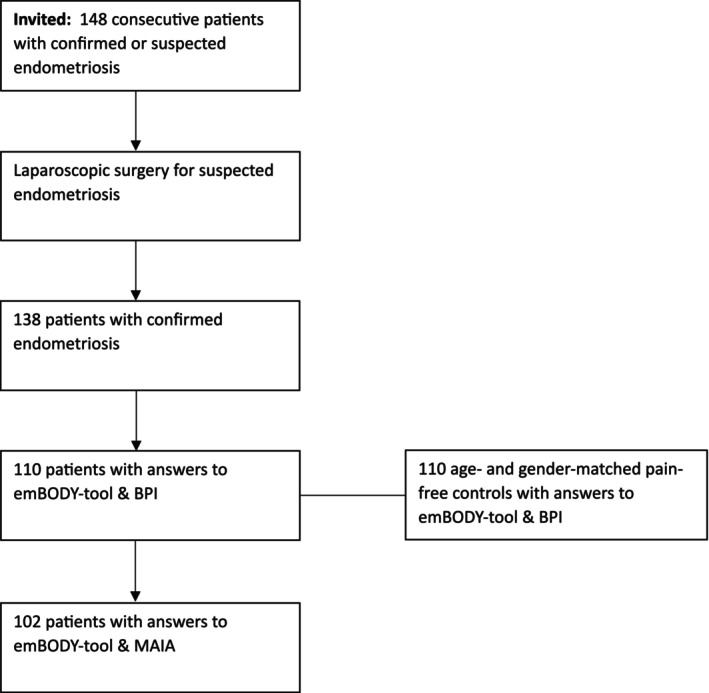
Flow chart demonstrating the inclusion of the final sample of endometriosis patients. Age‐ and gender‐matched pain‐free controls were selected from a larger population sample adjusted for the patient cohort of the current study. emBODY‐tool = computer‐based, topographical self‐report method to produce emotional, pain and sensitivity maps. BPI = Brief Pain Inventory. MAIA = Multidimensional Assessment of Interoceptive Awareness.

Figure 1 shows the flow chart for the study. During laparoscopic surgery, endometriotic lesions were not found in ten patients who were then excluded from the main analyses.

In total, 110 endometriosis patients and their 110 age‐matched pain‐free controls completed the emBODY‐tool task. The patients did not differ from the pain‐free controls in age but had higher BMIs than controls (U = 4955, *p* = 0.02). Pain‐free controls had higher educational levels than patients (ꭓ^2^ = 35.264, df = 3, *p* < 0.001). Demographic information for patients and controls is shown in Table 1.

**TABLE 1:** Demographic information for endometriosis patients and healthy controls.

	Patient *n* = 110^a^	Control *n* = 110^a^	*p* ^b^
Age	35 (29, 41)	33 (25, 42)	0.19
BMI	25.5 (22.6, 29.0)	23.4 (21.6, 27.0)	0.02
Education			< 0.001
Primary school	0 (0.0%)	4 (3.6%)	
Secondary school	23 (21.0%)	11 (10.0%)	
High school	53 (48.0%)	22 (20.0%)	
University or higher	34 (31.0%)	73 (66.4%)	
Migraine			0.001
Yes	36 (32.7%)	14 (12.7%)	
Headache			< 0.001
Yes	45 (40.9%)	20 (18.2%)	
Abdominal pain			< 0.001
Yes	102 (92.7%)	0 (0.0%)	
Dysmenorrhoea			< 0.001
Yes	83 (75.5%)	0 (0.0%)	
Back/shoulder pain			< 0.001
Yes	81 (73.6%)	47 (42.7%)	
Joint/limb pain			0.007
Yes	45 (40.9%)	24 (21.8%)	
OTC analgesics			< 0.001
Daily	16 (14.5%)	0 (0.0%)	
Weekly	36 (32.7%)	10 (9.1%)	
Monthly	42 (38.2%)	41 (37.3%)	
Rarely	14 (12.7%)	56 (50.9%)	
Never	2 (1.8%)	3 (2.7%)	
Prescribed analgesics			< 0.001
Daily	20 (18.2%)	0 (0.0%)	
Weekly	27 (24.5%)	3 (2.7%)	
Monthly	25 (22.7%)	7 (6.4%)	
Rarely	22 (20.0%)	44 (40.0%)	
Never	16 (14.5%)	56 (50.9%)	

Abbreviations: BSM, Bodily Sensation Map; MAIA, The Multidimensional Assessment of Interoceptive Awareness; OTC, over‐the‐counter.

^a^ Median (IQR); *n* (%).

^b^ Wilcoxon rank sum test; Fisher's exact test; Pearson's Chi‐squared test.

### 3.1 Pain and Self‐Reported Emotional Experience in Patients and Pain‐Free Controls

Descriptive information on current self‐reported mood, basic emotional states, as well as pain intensity and interference on the BPI for patients and pain‐free controls, are shown in Table 2. Patients and pain‐free controls did not differ on self‐reported anxiety or depression (p's 1.000). Patients and pain‐free controls differed on all BPI scales (all *p*'s < 0.001), with patients reporting significantly more pain intensity and interference.

**TABLE 2:** Descriptive information on self‐reported depression, anxiety, basic emotional states, as well as pain intensity and interference separately for patients and pain‐free controls. Answers to Brief Pain Inventory (BPI) were available for *n* = 156 participants; *p*‐values are Holm‐Bonferroni‐corrected for multiple comparisons.

	Patient *n* = 110/96^a^	Control *n* = 110/60^a^	*p* ^b^
Mood/basic emotional state			
Depression	1 (0, 3)	0 (0, 2)	1.000
Anxiety	1 (0, 3)	1 (0, 2)	1.000
Happiness	5 (3, 6)	6 (3, 7)	0.368
Sadness	0 (0, 2)	0 (0, 1)	1.000
Anger	0 (0, 0)	0 (0, 0)	0.644
Fear	1 (0, 4)	0 (0, 2)	0.037
Surprise	0 (0, 1)	0 (0, 1)	0.908
Disgust	0 (0, 0)	0 (0, 1)	0.037
Brief Pain Inventory (BPI)^c^			
Pain intensity	2.75 (1.50, 4.75)	1.25 (1.00, 1.50)	< 0.001
Pain interference	2.43 (0.71, 4.86)	0.43 (0.14, 1.43)	< 0.001
Activity interference	2.38 (0.75, 4.75)	0.50 (0.00, 1.13)	< 0.001
Affective interference	2.33 (0.67, 5.17)	0.33 (0.00, 0.67)	< 0.001

^a^ Median (Q1, Q3).

^b^ Wilcoxon rank sum test.

^c^ *n* = 156.

In a two‐way within‐between ANOVA on trimmed means, with a between‐subject factor as group membership (two levels: patient and healthy control) and within‐subject factor as basic emotional state (eight levels), there was a significant main effect of current emotional state (F(7, 93.73) = 113.08, *p* < 0.001), but no differences between the groups (F(1, 128.22) = 0.16, *p* = 0.69). The interaction between group and emotional state was also not significant (F(7, 93.73) = 1.37, *p* = 0.226). In a complementary analysis, the groups differed in the perception of fear (U = 4804.5, corrected *p* = 0.037) and disgust (U = 6949, corrected *p* = 0.037). Patients felt more fear (mean = 2.14, SD = 2.68) than did pain‐free controls (mean = 1.15, SD = 1.8), but less disgust (mean = 0.34, SD = 1.27) than controls (mean = 0.49, SD = 1.13).

### 3.2 Pain and Interoception in the Patients

Approximately 45% of the patients evaluated their current pain to be ≥ 3/10, and approximately 66% evaluated their pain on average during the last 24 h to be ≥ 3/10. Table 3 shows the correlations between BPI and MAIA subscales in endometriosis patients. The higher the patients scored on the MAIA subscale *Emotional awareness*, the more pain intensity they reported (r_s_ = 0.27, corrected *p* = 0.036). On the other hand, higher scores on MAIA subscale *Trusting* associated with less reported affective interference (r_s_ = −0.35, corrected *p* = 0.002).

**TABLE 3:** Correlations between Brief Pain Inventory (BPI) and interoceptive awareness (MAIA) in endometriosis patients (*n* = 102).

BPI	Pain intensity	Pain interference	Activity interference	Affective interference
MAIA				
Noticing	0.10	0.06	0.07	0.06
Not‐worrying	−0.18	−0.18	−0.09	−0.25^a^
Attention regulation	−0.02	−0.05	−0.02	−0.07
Emotional awareness	0.27^b^	0.21^a^	0.23^a^	0.21^a^
Self‐regulation	0.04	−0.00	0.06	−0.04
Body listening	0.04	−0.01	0.05	−0.03
Trusting	−0.20	−0.25^a^	−0.14	−0.35^b^

Abbreviations: BSM, Bodily Sensation Map; MAIA, The Multidimensional Assessment of Interoceptive Awareness.

^a^ Holm‐Bonferroni‐corrected *p* ≤ 0.10.

^b^ Holm‐Bonferroni‐corrected *p* ≤ 0.05.

### 3.3 Emotional and Sensory Body Maps, and Interoceptive Awareness in the Patients

Regarding the associations between BSMs and interoceptive awareness in the patients, we found positive correlations between emotional body maps for happiness, sadness, and anger with certain MAIA subscales (Table 4). Higher scores in the MAIA subscale *Body listening* correlated with more colouring for sadness (r_s_ = 0.30, corrected *p* = 0.015). In addition, the higher the patients scored in the MAIA subscales *Self‐regulation* and *Body listening*, the more they coloured in for happiness in the BSMs (for *Self‐regulation*: r_s_ = 0.28, corrected *p* = 0.027, for *Body listening*: r_s_ = 0.31, corrected *p* = 0.011). Finally, the higher the patients scored in the MAIA subscale *Emotional awareness*, the more they coloured in for anger (r_s_ = 0.31, corrected *p* = 0.013).

**TABLE 4:** Correlations between basic emotions (BSM) and interoceptive awareness (MAIA) in endometriosis patients (*n* = 102).

BSM	Sadness	Happiness	Anger	Surprise	Fear	Disgust	Neutral
MAIA							
Noticing	0.16	0.23	0.16	0.09	0.17	0.16	0.01
Not‐worrying	−0.01	0.07	−0.02	−0.04	0.06	−0.02	−0.04
Attention regulation	0.02	0.11	0.11	0.10	0.03	0.08	−0.07
Emotional awareness	0.18	0.23	0.31^b^	0.19	0.13	0.17	0.03
Self‐regulation	0.14	0.28^b^	0.10	0.11	0.04	−0.01	0.05
Body listening	0.30^b^	0.31^b^	0.23^a^	0.16	0.19	0.06	0.14
Trusting	0.11	0.26^a^	0.08	−0.05	−0.05	0.01	−0.10

Abbreviations: BSM, Bodily Sensation Map; MAIA, Multidimensional Assessment of Interoceptive Awareness.

^a^ Holm‐Bonferroni‐corrected *p* ≤ 0.10.

^b^ Holm‐Bonferroni‐corrected *p* ≤ 0.05.

Table 5 shows how pain and sensitivity maps are associated with interoceptive awareness in the patients. The MAIA subscale *Emotional awareness* correlated positively with the areas coloured in for persistent pain (r_s_ = 0.26, corrected *p* = 0.038), while the MAIA subscale *Trusting* correlated negatively with the areas coloured in for current pain (r_s_ = −0.29, corrected *p* = 0.015). In addition, MAIA subscales *Trusting* and *Not‐worrying* correlated positively with colouring for hedonic sensitivity (for *Not‐worrying* r_s_ = 0.34, corrected *p* = 0.002, for *Trusting* r_s_ = 0.25, corrected *p* = 0.045).

**TABLE 5:** Correlations between pain and sensitivities (BSM), and interoceptive awareness (MAIA) in endometriosis patients (*n* = 102).

BSM	Current pain	Persistent pain	Tactile sensitivity	Nociceptive sensitivity	Hedonic sensitivity
MAIA					
Noticing	0.04	0.16	−0.07	0.08	0.16
Not‐worrying	−0.21	−0.11	0.02	−0.09	0.34^b^
Attention regulation	0.04	0.12	−0.02	0.08	0.17
Emotional awareness	0.18	0.26^b^	0.00	0.23^a^	0.08
Self‐regulation	−0.11	0.03	−0.14	−0.15	0.16
Body listening	0.02	0.19	0.01	0.06	0.19
Trusting	−0.29^b^	−0.01	0.04	−0.15	0.25^b^

Abbreviations: BSM, Bodily Sensation Map; MAIA, Multidimensional Assessment for Interoceptive Awareness.

^a^ Holm‐Bonferroni‐corrected *p* ≤ 0.10.

^b^ Holm‐Bonferroni‐corrected *p* ≤ 0.05.

### 3.4 Bodily Sensations in Patients and Pain‐Free Controls

**FIGURE 2:**
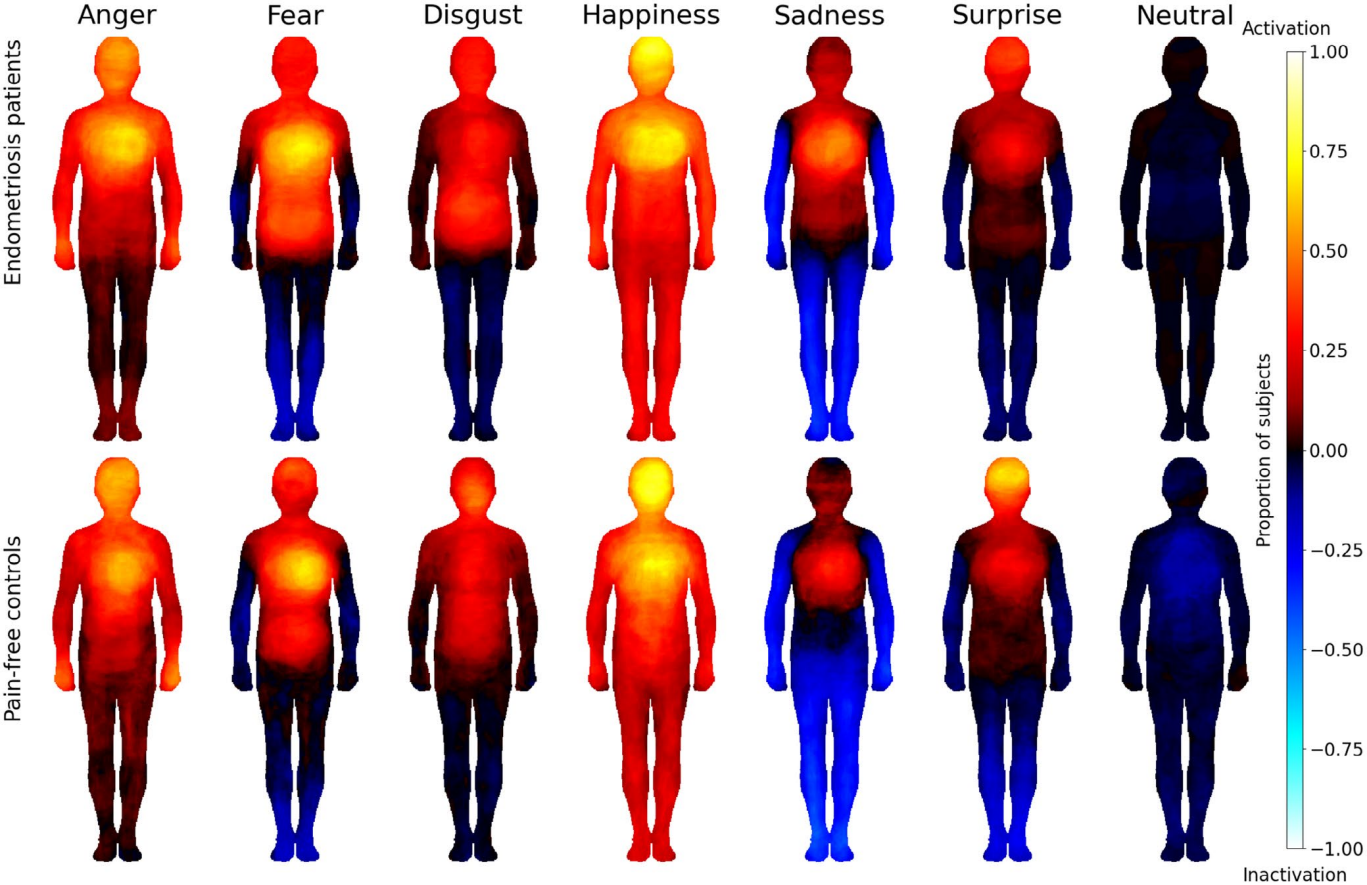
Bodily maps for basic emotions in patients (top row *n* = 110) and pain‐free controls (bottom row *n* = 110). Hot colors indicate higher activity.

Figure 2 shows the bodily maps for the basic emotions and the neutral state for patients and pain‐free controls. No pixel‐wise differences were found in the BSMs between patients and pain‐free controls. We also compared the extent (number of coloured pixels) of self‐reported emotional experience in patients and controls. In a two‐way within‐between ANOVA on trimmed means with a between‐subject factor as group membership (two levels: patient and healthy control) and within‐subject factor as BSM for emotion (seven levels), there was a significant main effect of emotion (F(6, 97.98) = 69.46, *p* < 0.001), but no significant effect of group (F(1, 127.15) = 0.79, *p* = 0.375). The interaction between group and emotion was not significant, either (F(6, 97.98) = 1.30, *p* = 0.264). In a complementary analysis, most of the contrasts were insignificant (corrected *p*'s = 1.0) except for surprise (U = 7334, corrected *p* = 0.026) and neutral (U = 7524, corrected *p* < 0.001) emotional states. Patients coloured in for less surprise in the body maps (mean = 0.15, SD = 0.17) than pain‐free controls (mean = 0.19, SD = 0.16). In addition, patients coloured less for a neutral emotional state (mean = 0.04, SD = 0.1) than controls (mean = 0.11, SD = 0.19).

**FIGURE 3:**
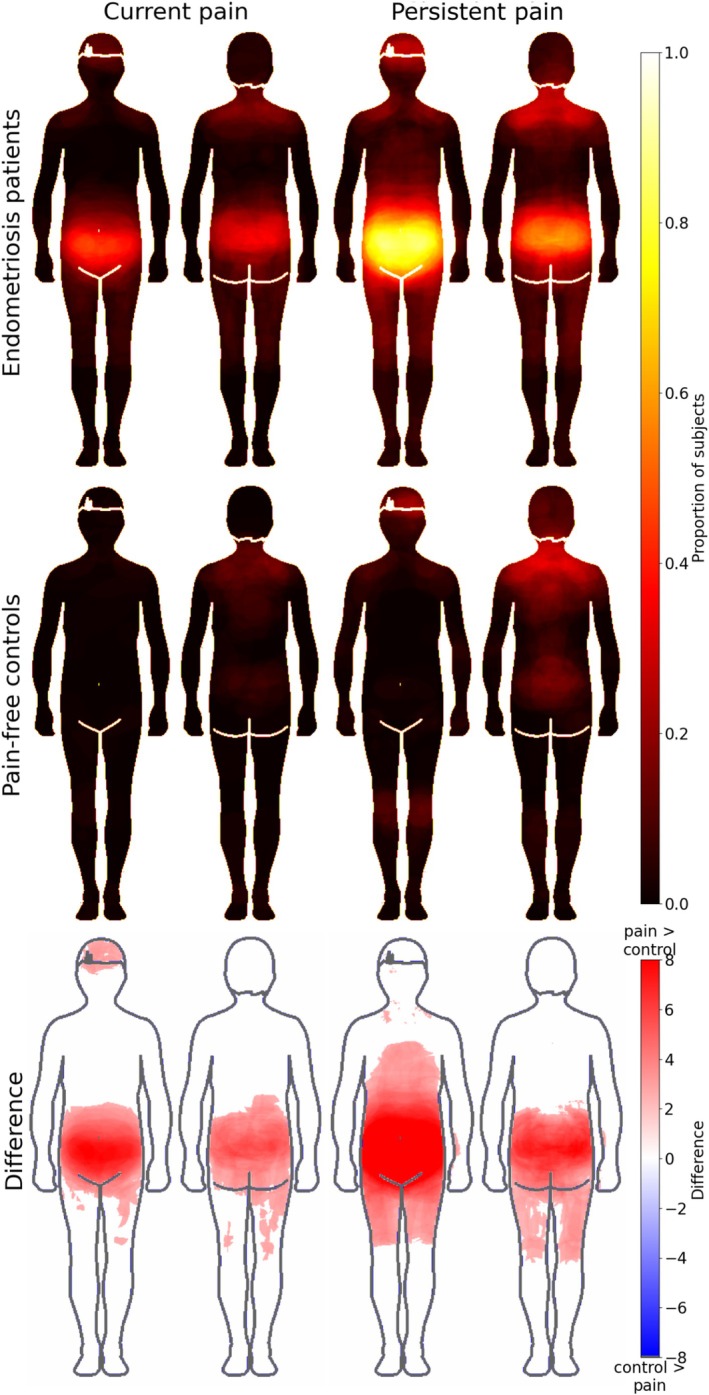
Bodily maps for current (two leftmost columns) and persistent pain (two rightmost columns) in patients and healthy controls. Endometriosis patients (top row, *n* = 108) reported significantly more current and persistent pain than the control group (middle row, *n* = 110). The bottom row shows regions where patients reported stronger pain than controls. The front side of the body is always shown on the left. The data on the bottom row are thresholded at *p* < 0.05, FDR‐corrected.

Figure 3 shows the pain maps for patient and control groups. Patients coloured in more areas (mean = 0.12, SD = 0.1) than controls (mean = 0.03, SD = 0.05) (U = 9796.5, *p* < 0.001). Specifically, patients coloured more for current pain (mean = 0.07) than controls (mean = 0.02) (U = 2957, corrected *p* < 0.001) and more for persistent pain (mean = 0.16) than pain‐free controls (mean = 0.05) (U = 1595, corrected *p* < 0.001).

**FIGURE 4:**
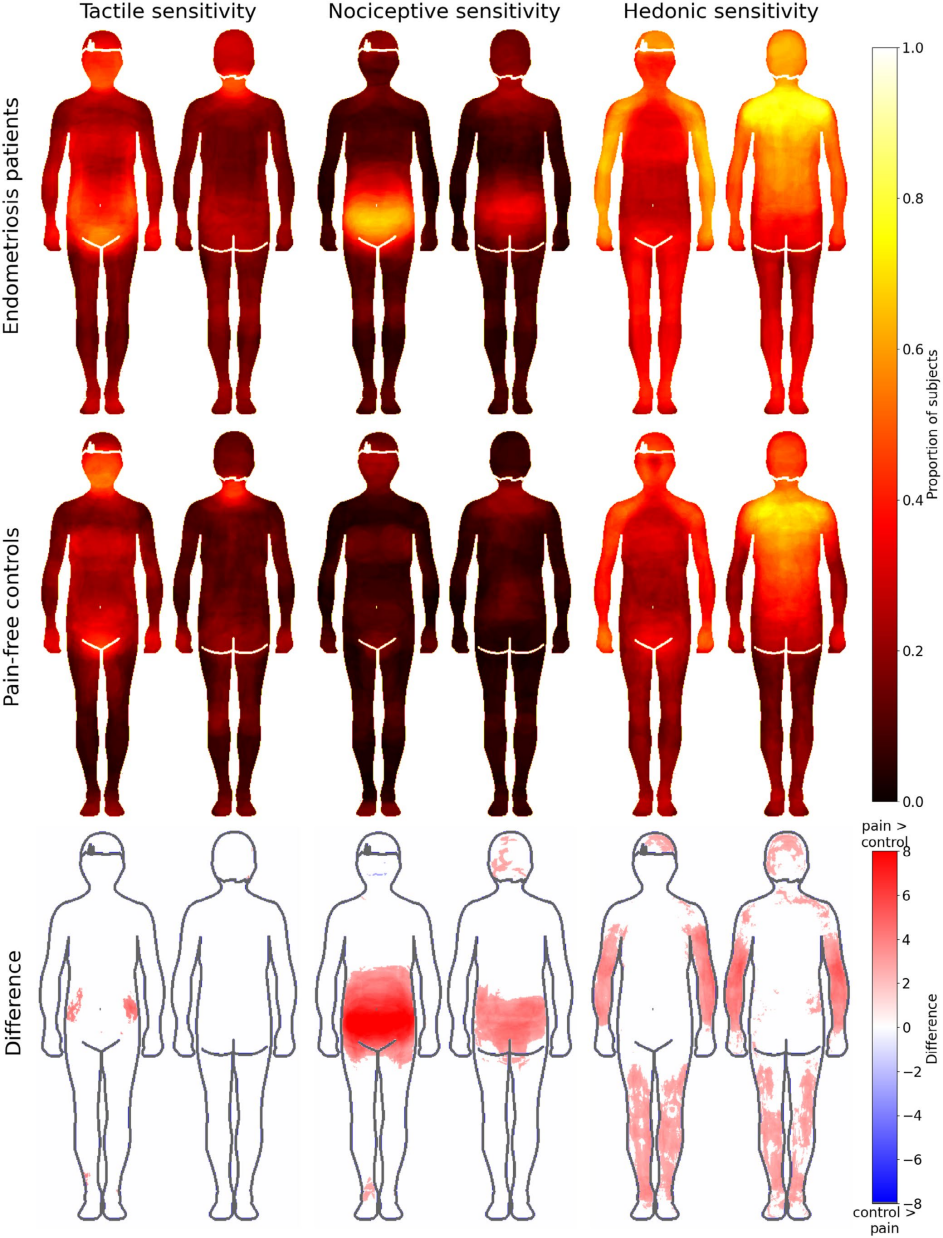
Bodily maps for tactile (two leftmost columns), nociceptive (two middle columns), and hedonic (two rightmost columns) sensitivities across the groups. Endometriosis patients (top row, *n* = 107) reported significantly more activation in nociceptive and hedonic sensitivities than pain‐free controls (middle row, *n* = 110). The bottom row shows the pixel‐wise differences between the two groups. The front side of the body is always shown on the left. The data on the bottom row are thresholded at *p* < 0.05, FDR‐corrected.

Figure 4 shows the sensitivity maps for the patient and control groups. In a two‐way within‐between ANOVA on trimmed means with a between‐subject factor as group membership (two levels: patient and healthy control) and within‐subject factor as BSM for sensitivities (three levels), there was a significant main effect of group (F(1, 120.36) = 15.01, *p* < 0.001) and sensitivity (F(2, 98.23) = 49.51, *p* < 0.001). The interaction between group and sensitivity was not significant (F(2, 98.23) = 1.31, *p* = 0.275). Patients coloured in more areas (mean = 0.26, SD = 0.26) than controls (mean = 0.18, SD = 0.19) (U = 43,088, *p* < 0.001). Specifically, patients coloured on average more for nociceptive sensitivity (mean = 0.14), than controls (mean = 0.07) (U = 3936, corrected *p* < 0.001), and more for hedonic sensitivity (mean = 0.42) than controls (mean = 0.30) (U = 4684, corrected *p* = 0.008). No pairwise difference was found for tactile sensitivity.

### 3.5 Pain, Self‐Reported Emotional Experience, and Bodily Sensations in Patients and Pain‐Free Controls

**FIGURE 5:**
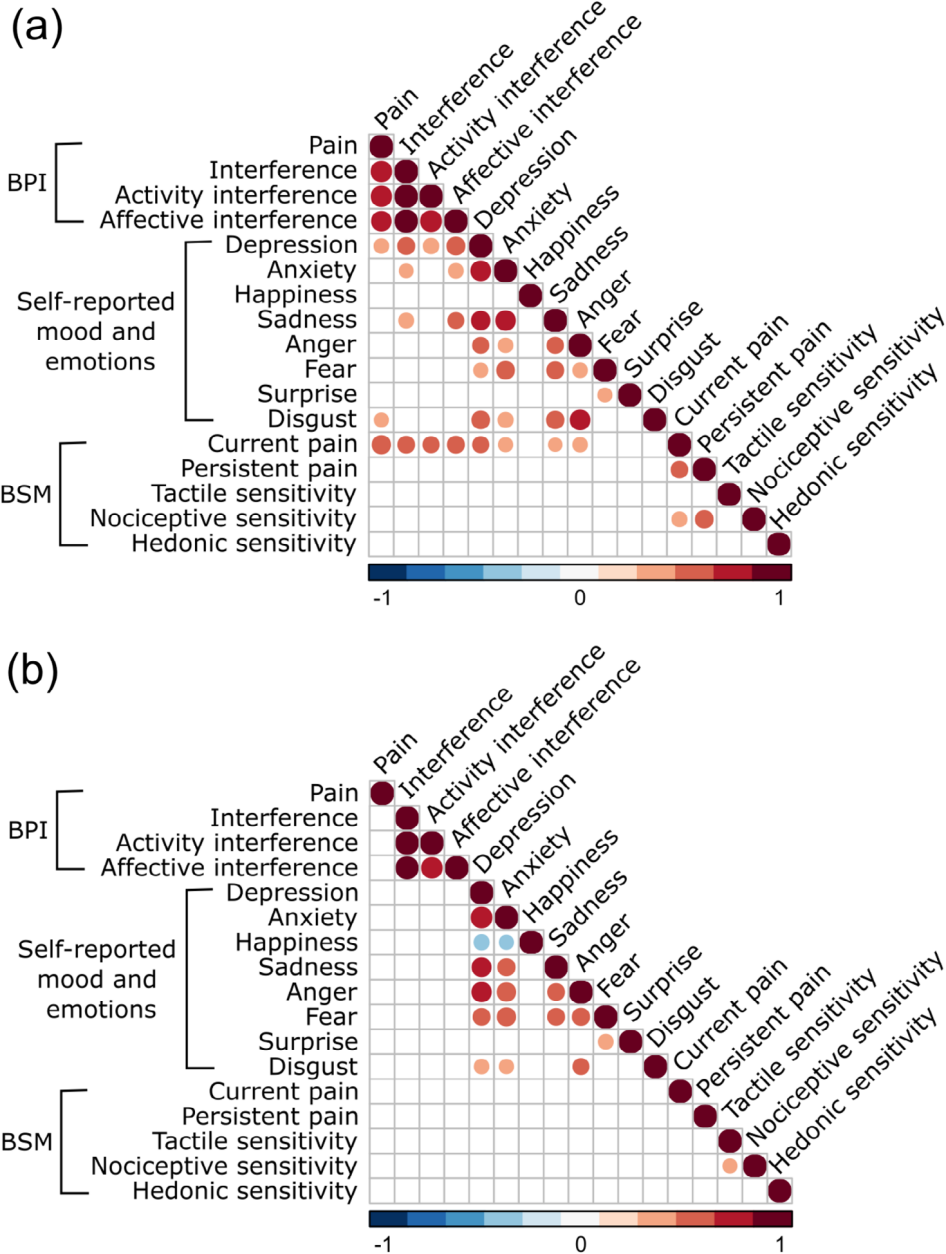
Correlation matrices for pain, self‐reported emotional experience, and bodily sensitivities in (a) endometriosis patients (*n* = 110) and (b) pain‐free controls (*n* = 110). Hot colours indicate positive correlations; cool colours indicate negative ones. Larger circles indicate stronger and smaller circles indicate weaker associations between variables. Only the Holm‐Bonferroni‐corrected significant associations are plotted (*p* < 0.05). BPI = Brief Pain Inventory. BSM = Bodily Sensation Map. All correlations are shown in Table S1 (for patients) and Table S2 (for pain‐free controls).

Figure 5 shows the correlation matrices for pain, self‐reported emotional experience, and bodily sensitivities in **(a)** endometriosis patients and **(b)** pain‐free controls. In patients (Figure 5a), higher pain intensity and interference (both activity and affective interference) were associated with more colouring for current pain in the BSMs for pain (r_s_'s ranging 0.48–0.62 and all corrected *p'*s < 0.001). In addition, self‐reported sadness, anger, anxiety, and depression were associated with more colouring for current pain in patients' pain maps (r_s_'s ranging 0.33–0.48 and corrected *p*'s < 0.001–0.024). In pain‐free controls (Figure 5b), no significant associations between pain, self‐reported emotions and the colouring of BSMs for pain and sensitivities were found. In addition, we found no significant associations between pain intensity or pain interference (BPI) and BSMs for emotions either in patients or healthy controls. Specific correlation coefficients for pain, self‐reported emotional experience and bodily sensitivities in patients and pain‐free controls are shown in Table S1 and Table S2.

## 4 Discussion

We were interested in how interoception, emotional processing, and pain interact with each other in endometriosis patients, and how patients with painful endometriosis might differ from healthy controls in their ways of experiencing bodily sensations including emotions. We found that, in endometriosis patients, interoceptive features relating to *Emotional awareness* (acknowledging the connection between bodily sensations and emotions) were associated with negative pain‐related factors. On the other hand, interoceptive features relating to *Trusting* (finding one's body safe and trustworthy) were associated with positive pain‐related factors. Endometriosis patients did not differ from healthy participants in their ways of colouring basic emotions but did report more current fear than healthy controls. Finally, patients reported more current and persistent pain, as well as nociceptive sensitivity, and, interestingly, also more hedonic sensitivity than healthy controls.

### 4.1 Pain

All patients had painful endometriosis requiring surgery. As expected, they coloured statistically significantly more for both current and persistent pain and nociceptive sensitivity than controls in the BSMs. The colouring for current and persistent pain and nociceptive sensitivity was most intense in the pelvic area. For persistent pain, the patients also coloured significantly stronger in a larger area, including thighs and stomach, than pain‐free controls.

Pain is a key factor causing disability in endometriosis. Inflammation with peripheral and central sensitisation are essential features of pain in endometriosis (Bulun et al. 2019; Evans et al. 2007; Lousse et al. 2012; Zondervan et al. 2020). Currently, endometriosis is considered as a chronic systemic disease that may affect several organ functions and be associated with anxiety, depression, and fatigue (Taylor et al. 2021). These mechanisms can explain the fact that over 30% of the endometriosis patients in our cohort reported a history of migraine, and over 70% a history of back or shoulder pain. Our findings are in line with a recent study reporting that endometriosis patients were twice as likely to have migraine and back pain problems than women without endometriosis (Rossi et al. 2023).

### 4.2 Emotions and Pain

Endometriosis patients and pain‐free controls did not differ in their self‐reported anxiety or depression. In endometriosis patients, but not in controls, higher self‐reported anxiety, depression, sadness, and anger were associated with more colouring for current pain in the pain maps.

Endometriosis patients and healthy controls did not differ significantly in their self‐reported evaluations on current emotional experience overall. However, in pairwise comparisons, endometriosis patients reported more current fear than controls. This may be related to the fact that pain sensitises to fearful stimuli and facilitates fear learning (Simons et al. 2014). Also, the fact that the patients were about to have a medical procedure could have contributed to their experiencing more fear than controls.

### 4.3 Interoception and Pain

There is growing interest in studying the associations between interoception and persistent pain (Di Lernia et al. 2016). In prolonged pain and its maintenance, altered interoceptive processes may facilitate learned avoidance of internal stimuli that are associated with pain, reinforcing fearful interpretations of bodily sensations (Gnall et al. 2024).

Endometriosis patients who expressed high *Emotional awareness* also reported higher pain intensity and coloured larger areas for persistent pain in the BSMs. On the other hand, patients who expressed high *Trusting*, reported less affective pain interference and coloured fewer areas for current pain. In addition, patients who expressed high *Not‐worrying* coloured more areas for hedonic sensitivity in the BSMs. Our findings are in line with earlier research suggesting that interoception may play either an adaptive or a maladaptive role in pain perception and control (Ciaramella et al. 2023; Colgan et al. 2022; Hanley et al. 2017; Mehling et al. 2009).

The MAIA questionnaire measures different modes of attention towards bodily sensations with the aim of distinguishing between adaptive and maladaptive forms of interoceptive attention (Mehling et al. 2013). For example, *Emotional awareness* relates to mind–body integration, where awareness of certain physical sensations is the sensory aspect of emotions, the “somatic markers” (Mehling et al. 2012). Mehling et al. (2012) suggested that more awareness of how body sensations correspond to emotional states, without the ability to use self‐regulatory skills to reduce distress from those sensations, may increase anxiety and affect pain perception. On the other hand, trusting bodily sensations and feeling one's body safe and trustworthy makes it possible to perceive and process bodily sensations without anxiety‐driven interpretations and use those sensations for decision‐making in a more neutral way. Earlier research on interoceptive interventions for chronic pain have revealed promising results on mind–body therapies that emphasise attending towards and accepting bodily sensations (Mehling et al. 2024; Voss et al. 2023). It has been suggested that turning towards, instead of distracting oneself from uncomfortable sensations, enhances self‐efficacy and self‐regulatory capacities, both important constructs in pain management.

The endometriosis patients in our study scored significantly lower in all MAIA subscales (*p*'s < 0.001) than healthy participants in the developmental study of the MAIA questionnaire (Mehling et al. 2012). When compared with low back pain patients (Mehling et al. 2013), the patients in our study expressed significantly higher *Not‐distracting*, but significantly lower *Self‐regulation* and *Trusting* (*p*'s < 0.001). These findings suggest that trusting one's body and being able to regulate the distress caused by sensations from the body may be particularly challenging for endometriosis patients.

### 4.4 Bodily Sensation Maps

In general, endometriosis patients and pain‐free controls expressed emotions in the emBODY‐tool in a similar fashion. Endometriosis patients coloured statistically significantly more pain than controls. More interestingly, the patients also coloured more nociceptive and hedonic sensitivities than controls, whereas there was no difference in tactile sensitivities.

Our findings agree in part with another recent study using the emBODY‐tool in chronic pain patients (Ojala et al. 2023), which also showed that patients reported more nociceptive sensitivity than pain‐free controls. Ojala et al. (2023) also reported an overall reduced embodied experience of emotions in patients than controls. The endometriosis patients in our study reported more hedonic sensitivity than controls, unlike the patients in the study by Ojala et al. (2023), which included both genders and a mixture of chronic pain conditions.

One potential explanation for the differences between the two studies could be that the endometriosis patients crave more social support and stress buffering, due to the intimate nature of the condition. Earlier research showed that hedonic sensitivity maps are associated with social touch (Suvilehto et al. 2015). Social touch is a key aspect in establishing and maintaining social relationships (Suvilehto et al. 2023) and can promote subjective well‐being via multiple pathways (Jakubiak and Feeney 2017).

Our findings regarding pain, emotions, interoception, and bodily sensations in endometriosis patients may be interpreted through the construct of interoceptive fear‐learning (De Peuter et al. 2011). The fact that, compared with controls, endometriosis patients expressed more current fear and clear associations between certain interoceptive features and experience of pain, may be related to a tendency to connect interoceptive information to painful bodily experiences, potentially maintaining the hypervigilance towards even neutral bodily sensations (De Peuter et al. 2011; Eccleston and Crombez 1999). In addition, interoceptive fear‐learning may manifest in avoidance of interoceptive information that could be interpreted as a risk for recurring pain, a mechanism which might be especially strong in visceral or fluctuating pain (De Peuter et al. 2011), as in endometriosis.

### 4.5 Strengths and Limitations

The main strength of this study is the multifaceted information it provides about psychological factors relating to endometriosis patients. In addition, both the emBODY‐tool and the MAIA‐questionnaire are relatively new methods of describing and operationalising subjective emotive and bodily experiences in pain patients. We used these methods together to provide a comprehensive understanding of the inner experiences of endometriosis patients.

This study has limitations. Firstly, this is a cross‐sectional study, and only correlational analyses were possible. Prospective studies are needed to analyse possible causal effects. Secondly, we used numerical rating scales for anxiety and depression instead of a standardised assessment tool. Thirdly, the fact that the controls used their own devices for colouring the body maps instead of a standardised setup (as with the patients), may have caused more variability in the colourings of the pain‐free controls than with endometriosis patients. Furthermore, the patients were anticipating surgery during the data collection, which might have affected their emotional state and therefore the generalisability of the results. Finally, all study subjects were female, necessitating future studies in other pain conditions in both genders.

## 5 Conclusions

Our findings suggest interoceptive features related to trusting one's body and finding it safe, as well as not worrying about unpleasant physical sensations, to be the most relevant in adaptive pain regulation, whereas heightened awareness of one's emotions in the body may be associated with more intensively experienced pain. These findings provide new perspectives for the targeting of psychosocial treatments for endometriosis patients, highlighting the importance of interventions that focus on interoceptive performance so that the ability to calmly identify and relate to bodily sensations may increase. In addition, the high rate of comorbid pain conditions in endometriosis patients should be considered when planning comprehensive and adequate treatment.

Overall, our findings suggest that endometriosis patients may have a distinctive footprint of pain, and a combination of different psychological factors associated with their bodily experiences and well‐being.

## Author Contributions

S.P. drafted the manuscript and J.S., P.H., O.H., R.S., and E.K. revised it critically for important intellectual content. E.K., R.S., P.H., and O.H. designed the study. P.H. and O.H. executed data collection. S.P. analysed the data with guidance from J.S., who was also responsible for the analysis of the Bodily Sensation Maps. S.P., J.S., E.K., and R.S. interpreted the analysed data. E.K. supervised the project. All contributing authors discussed the results, commented on the manuscript, and approved the final submitted version.

## Conflicts of Interest

S.P. has received a speaking honorarium from Gedeon Richter Nordics AB. The authors have no other conflicts of interest to declare regarding this work.

## Supporting information


**Table S1:** Correlations between pain, self‐reported depression, anxiety, six basic emotions, and bodily sensitivities in endometriosis patients (*n* = 110).

**Table S2:** Correlations between pain, self‐reported depression, anxiety, six basic emotions, and bodily sensitivities in pain‐free controls (*n* = 110).
