# Supplementary material for: Pain, Emotions, Interoception, and Bodily Sensations in Patients With Endometriosis Bodily Sensations in Patients With Endometriosis
Source: Eur J Pain. 2025 Oct 6;29(10):e70144. doi: 10.1002/ejp.70144 (PMC12500402; doi:10.1002/ejp.70144)
Supplement: Supplementary file 2 — Table S2: Correlations between pain, self‐reported depression, anxiety, six basic emotions, and bodily sensitivities in pain‐free controls (n = 110). [file EJP-29-0-s002.docx]

**Supplementary table 2.** Correlations between pain, self-reported depression, anxiety, six basic emotions, and bodily sensitivities in pain-free controls (n = 110).

|  | **BPI** | | | | **Self-reported mood & emotions** | | | | | | | | **BSM sensitivities** | | | | |
| --- | --- | --- | --- | --- | --- | --- | --- | --- | --- | --- | --- | --- | --- | --- | --- | --- | --- |
|  | Pain | Interference | Activity interference | Affective interference | Depression | Anxiety | Happiness | Sadness | Anger | Fear | Surprise | Disgust | Current pain | Persistent pain | Tactile | Nociceptive | Hedonic |
| Pain | 1.00 |  |  |  |  |  |  |  |  |  |  |  |  |  |  |  |  |
| Interference | 0.39 | 1.00 |  |  |  |  |  |  |  |  |  |  |  |  |  |  |  |
| Activity interference | 0.37 | **0.97** **^b^** | 1.00 |  |  |  |  |  |  |  |  |  |  |  |  |  |  |
| Affective interference | 0.36 | **0.92 ^b^** | **0.79 ^b^** | 1.00 |  |  |  |  |  |  |  |  |  |  |  |  |  |
| Depression | -0.07 | 0.12 | 0.09 | 0.15 | 1.00 |  |  |  |  |  |  |  |  |  |  |  |  |
| Anxiety | -0.09 | 0.06 | -0.00 | 0.15 | **0.80^b^** | 1.00 |  |  |  |  |  |  |  |  |  |  |  |
| Happiness | 0.15 | 0.08 | 0.08 | 0.06 | **-0.42^b^** | **-0.36^a^** | 1.00 |  |  |  |  |  |  |  |  |  |  |
| Sadness | 0.00 | 0.23 | 0.19 | 0.26 | **0.67^b^** | **0.55^b^** | -0.10 | 1.00 |  |  |  |  |  |  |  |  |  |
| Anger | -0.17 | 0.17 | 0.13 | 0.21 | **0.70^b^** | **0.63^b^** | -0.19 | **0.52^b^** | 1.00 |  |  |  |  |  |  |  |  |
| Fear | -0.01 | 0.07 | 0.02 | 0.13 | **0.53^b^** | **0.62^b^** | -0.04 | **0.53^b^** | **0.55^b^** | 1.00 |  |  |  |  |  |  |  |
| Surprise | 0.03 | -0.12 | -0.12 | -0.09 | 0.14 | 0.15 | 0.16 | 0.12 | 0.11 | **0.40^b^** | 1.00 |  |  |  |  |  |  |
| Disgust | 0.02 | 0.20 | 0.15 | 0.24 | **0.39^a^** | **0.37^a^** | -0.15 | 0.23 | **0.47^b^** | **0.33^a^** | 0.18 | 1.00 |  |  |  |  |  |
| Current pain | 0.24 | 0.24 | 0.26 | 0.19 | 0.22 | 0.21 | -0.20 | 0.15 | 0.11 | 0.06 | -0.04 | 0.25 | 1.00 |  |  |  |  |
| Persistent pain | -0.00 | 0.18 | 0.17 | 0.17 | 0.16 | 0.01 | -0.15 | 0.16 | 0.01 | -0.03 | -0.06 | 0.06 | 0.28 | 1.00 |  |  |  |
| Tactile | -0.02 | 0.17 | 0.16 | 0.17 | -0.04 | 0.00 | 0.02 | -0.00 | -0.11 | -0.05 | 0.11 | 0.05 | 0.11 | 0.09 | 1.00 |  |  |
| Nociceptive | 0.13 | -0.02 | -0.06 | 0.04 | 0.02 | 0.05 | -0.07 | -0.00 | -0.07 | -0.01 | -0.06 | 0.04 | 0.03 | 0.13 | **0.36^a^** | 1.00 |  |
| Hedonic | -0.02 | 0.14 | 0.09 | 0.20 | 0.17 | 0.25 | -0.15 | 0.18 | 0.00 | -0.03 | 0.07 | 0.06 | 0.16 | 0.13 | 0.31 | 0.16 | 1.00 |

BPI = Brief Pain Inventory
BSM sensitivities = Bodily Sensation Maps for pain and sensitivities
^a^ Holm-Bonferroni-corrected *p* <0.05
^b^ Holm-Bonferroni-corrected *p* <0.001
